# Genetic susceptibility, evolution and the kuru epidemic

**DOI:** 10.1098/rstb.2008.0087

**Published:** 2008-11-27

**Authors:** Simon Mead, Jerome Whitfield, Mark Poulter, Paresh Shah, James Uphill, Jonathan Beck, Tracy Campbell, Huda Al-Dujaily, Holger Hummerich, Michael P. Alpers, John Collinge

**Affiliations:** 1 MRC Prion Unit and Department of Neurodegenerative Disease, UCL Institute of Neurology, National Hospital for Neurology and Neurosurgery, Queen SquareLondon WC1N 3BG, UK; 2 Papua New Guinea Institute of Medical ResearchPO Box 60, Goroka, EHP 441, Papua New Guinea; 3 Centre for International Health, ABCRC, Shenton Park Campus, Curtin UniversityGPO Box U1987, Perth, WA 6845, Australia

**Keywords:** kuru, genetics, evolution

## Abstract

The acquired prion disease kuru was restricted to the Fore and neighbouring linguistic groups of the Papua New Guinea highlands and largely affected children and adult women. Oral history documents the onset of the epidemic in the early twentieth century, followed by a peak in the mid-twentieth century and subsequently a well-documented decline in frequency. In the context of these strong associations (gender, region and time), we have considered the genetic factors associated with susceptibility and resistance to kuru. Heterozygosity at codon 129 of the human prion protein gene (*PRNP*) is known to confer relative resistance to both sporadic and acquired prion diseases. In kuru, heterozygosity is associated with older patients and longer incubation times. Elderly survivors of the kuru epidemic, who had multiple exposures at mortuary feasts, are predominantly *PRNP* codon 129 heterozygotes and this group show marked Hardy–Weinberg disequilibrium. The deviation from Hardy–Weinberg equilibrium is most marked in elderly women, but is also significant in a slightly younger cohort of men, consistent with their exposure to kuru as boys. Young Fore and the elderly from populations with no history of kuru show Hardy–Weinberg equilibrium. An increasing cline in 129V allele frequency centres on the kuru region, consistent with the effect of selection in elevating the frequency of resistant genotypes in the exposed population. The genetic data are thus strikingly correlated with exposure. Considering the strong coding sequence conservation of primate prion protein genes, the number of global coding polymorphisms in man is surprising. By intronic resequencing in a European population, we have shown that haplotype diversity at *PRNP* comprises two major and divergent clades associated with 129M and 129V. Kuru may have imposed the strongest episode of recent human balancing selection, which may not have been an isolated episode in human history.

## 1. Introduction

Prion diseases or the transmissible spongiform encephalopathies are fatal neurodegenerative conditions, including Creutzfeldt–Jakob disease (CJD) in man and sheep scrapie (Collinge 2001). The central molecular event in prion replication is the post-translational recruitment of the normal neuronal prion protein (PrP^C^) into a self-propagating conformational isomer that accumulates as aggregated material (PrP^Sc^). This concept, termed the protein-only hypothesis, is supported by compelling experimental data (Prusiner 1998; Collinge & Clarke 2007).

Kuru came to the attention of Western medicine in the 1950s, as the affected area of the Eastern Highlands (EHP) of Papua New Guinea came under external administrative control. The Fore and neighbouring linguistic groups occupied a remote highland area that had had no direct contact with the outside world before the 1950s. Typically manifesting as a progressive ataxia, kuru had a dramatic impact on the Fore and provides our major experience of epidemic human prion disease (Gajdusek & Zigas 1957). It has attracted recent interest owing to the occurrence of variant CJD (vCJD), the human form of bovine spongiform encephalopathy, to which there has been a wide dietary population exposure in the UK and other European countries (Collinge 1999).

Fore kinship groups consumed deceased relatives at mortuary feasts, resulting in human–human prion transmission, although males over the ages of 6–8 years participated little, such that kuru, at its peak, predominantly affected adult females and children. Kuru was restricted to the Fore linguistic group and their immediate neighbours with whom they intermarried. From oral history, the first cases are dated to the early twentieth century, and thereafter increased in incidence. A peak annual mortality of over 2 per cent was recorded in some Fore villages. Some villages became largely devoid of young women (Zigas & Gajdusek 1959). Kuru has progressively disappeared from the younger Fore, consistent with the cessation of endocannibalism by 1960 (Alpers 2005). Established risk factors for kuru thus include gender, region and date of birth, these acting through their associations with participation in mortuary feasts and likely exposure to kuru-infected tissue.

Human and animal prion diseases are under strong genetic control (Mead 2006). A coding polymorphism at codon 129 of *PRNP* is a strong susceptibility factor for human prion diseases. Methionine homozygotes comprise 37 per cent of the UK population whereas valine homozygotes comprise 12 per cent, with 51 per cent heterozygotes (Palmer *et al*. 1991). Homozygosity at *PRNP* codon 129 predisposes to iatrogenic (Collinge *et al*. 1991) and sporadic CJD (Palmer *et al*. 1991) and results in a younger age at onset in some inherited prion diseases (Dlouhy *et al*. 1992; Poulter *et al*. 1992; Mead *et al*. 2006, 2007). In iatrogenic CJD caused by an exposure to contaminated pituitary hormones, heterozygotes have a longer mean incubation period than homozygotes (Brown *et al*. 2000). All cases of vCJD to date have been methionine homozygotes, and homozygotes of either allele have an earlier age of onset for kuru (Cervenakova *et al*. 1999). Heterozygosity at a different *PRNP* polymorphism, E219K, is also associated with a resistance to sporadic CJD in Japan (Shibuya *et al*. 1998). Heterozygosity is thought to confer resistance to prion disease by inhibiting homologous protein–protein interactions (Palmer *et al*. 1991).

In this review, we consider the genetic epidemiology of kuru and the evidence for non-neutral evolution of *PRNP* in EHP and elsewhere.

## 2. Analysis of the *PRNP* coding region in Papua New Guinea

The coding region of *PRNP* has been analysed in (i) paediatric and (ii) adult kuru sampled around the peak of the epidemic (Cervenakova *et al*. 1999), (iii) recent elderly kuru patients with a long incubation time (Collinge *et al*. 2006), (iv) healthy modern-day young Fore, (v) neighbouring linguistic groups of EHP with low or no documented kuru, (vi) elderly men and (vii) women who attended mortuary feasts but have not developed kuru, sampled in the past 15 years (Mead *et al*. 2003). These groups may be stratified *a priori* in terms of their likely susceptibility to kuru. The interpretation of kuru patients' susceptibility is complicated by uncertainty about the time of exposure, as mortuary feasts of deceased kuru patients took place over many years prior to the cessation of cannibalism in 1960. The mean incubation period of kuru has been estimated as approximately 12 years (Alpers 2008). Childhood or adolescent kuru would therefore reflect the most susceptible group as the incubation time is limited (to around the mean or shorter) by the age that children were old enough to participate in mortuary feasts. Healthy elderly women with multiple exposures at mortuary feasts reflect the most resistant group as they have clearly documented exposure but have proven disease free over many decades. Healthy elderly men may also be expected to be resistant to kuru but to a lesser degree than elderly women given their lower exposure. Elderly recent kuru patients with very long incubation times, over 50 years in some cases, are also likely to show genetic resistance given that their incubation time is much longer than the mean. Adult kuru patients sampled at the peak of the epidemic are difficult to classify in this way as their incubation time is uncertain. Also interesting is the possibility that the Fore population adapted to the kuru epidemic by inflation in the frequency of genetic resistance factors. The healthy young modern populations in EHP can be ranked by their overall exposure to kuru to consider this possibility.

*PRNP* codon 129 genotypes in groups of varying exposure and disease status are shown in table 1. A generality, based on cumulative evidence from human studies, is that individuals homozygous for *PRNP* codon 129 are susceptible to prion diseases whereas those heterozygous are resistant. Additionally, however, the homozygous genotypes 129MM and 129VV may show differential susceptibility; for example, all patients with vCJD have been 129MM, whereas early patients with iatrogenic CJD in the UK are particularly associated with codon 129VV (Collinge *et al*. 1991). The molecular basis for this susceptibility is complex, invoking the efficiency of heterologous protein–protein interactions, and conformational selection (Collinge 1999; Hill & Collinge 2003; Collinge & Clarke 2007): whether the infecting prion strain is a preferred or permissible conformation for PrP with 129M or 129V (Collinge *et al*. 1996; Wadsworth *et al*. 2004; Asante *et al*. 2006).

The stratified kuru groups show highly significant differences in codon 129 genotype frequencies. The most susceptible group, kuru children, is strongly associated with 129MM and to a lesser degree 129VV genotypes. The low susceptibility groups all show an excess of heterozygosity at codon 129 and particularly a deficit of 129MM. This finding is strongly significant in elderly women who have attended multiple mortuary feasts, but also very prominent in the small number of recent kuru patients with extremely long incubation times. The elevated heterozygosity in healthy old women is presumably caused by homozygous individuals having died of kuru prior to the group being sampled. Overall, these data support the inference of strong balancing selection acting on the Fore at *PRNP* concurrent with the kuru epidemic (Mead *et al*. 2003).

**Figure 1:**
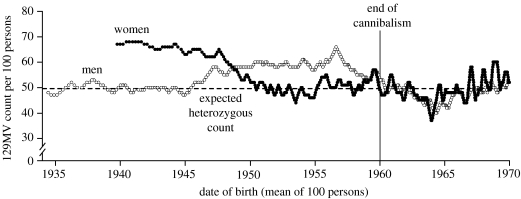
Heterozygosity at codon 129 in elderly men and women stratified by date of birth. This graph illustrates a significant excess of heterozygosity for both genders consistent with their exposure at mortuary feasts and the epidemiology of kuru. Males were exposed as infants at the peak of the epidemic in the 1950s whereas females were exposed throughout life. Excess heterozygosity of both genders is statistically significant in the relevant age cohorts.

Two issues are worthy of more detailed consideration. The exposure of the elderly male group occurred when they were young boys. Infant males were exposed to high-risk tissues (brain and spinal cord) at mortuary feasts in a similar way to females of all ages. Boys around the ages of 6–8 years would alter their participation in mortuary feasts, joining the adolescent and adult males, with low exposure to high-risk tissues. Assuming that these anthropological observations are correct, which is reasonable given their extensive corroboration (Whitfield *et al.* 2008), and that the *PRNP* codon 129 heterozygosity of elderly men gives an indication of their exposure as boys, we can explore the genetic data to estimate the likely exposure at mortuary feasts in the early twentieth century. This analysis is illustrated in figure 1. Here, we show the stratification of the heterozygosity of elderly men by ordering on date of birth and displaying a sliding window of heterozygosity from 100 male codon 129 genotypes. A peak of heterozygosity in the mid-1950s is consistent with epidemiological data regarding the peak incidence of kuru. These genetic data confirm oral history that kuru was a new disease in the twentieth century as extremely elderly men did not appear to be exposed to kuru as children.

A further issue of interest is whether the modern young Fore have a higher frequency of resistance genotypes than their neighbours in EHP, potentially as a consequence of the kuru epidemic in its recent history. It is important to consider the population consequences of heterozygote advantage over both homozygous genotypes. In this circumstance, a population may adapt to an equilibrium allele frequency determined by the relative fitness of the two homozygous genotypes. In kuru, 129MM is more susceptible than 129VV, which would be expected to result in equilibrium when 129V is the more frequent allele. 129V is more frequent in the Fore than its neighbours in EHP with no exposure to kuru; however, the difference is small (table 1). With rapid increases in allele frequency, one would expect to observe extensive linkage disequilibrium around the selected allele. In the Fore, the diversity of microsatellite alleles linked to 129V is not consistent with a large and rapid increase in 129V frequency from a low level (Mead *et al*. 2003). The population data are therefore consistent with some adaptation to kuru by an increase in 129V frequency in the Fore; however, it is also likely that 129V was a high-frequency allele in the EHP at the outset of the kuru epidemic.

## 3. The Fore in the context of a global analysis of PrP polymorphism

**Figure 2:**
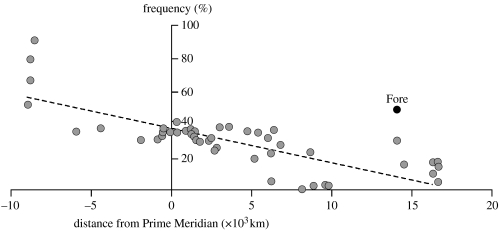
129V allele frequency in multiple populations here shown by distance from a longitudinal axis. The Fore linguistic group and other EHP populations are outstanding in this trend.

We and others have sequenced or genotyped many hundreds of individuals from populations selected to represent worldwide genetic diversity, in order to determine which *PRNP* nucleotides are polymorphic (here defined as an allele frequency greater than 0.01, at least two occurrences, in at least one population; Mead *et al*. 2003; Hardy *et al*. 2005; Soldevila *et al*. 2005). The results of these analyses are consistent. M129V is globally the most significant polymorphism with high derived allele (129V) frequency in Europe, and a reducing frequency into Africa or Asia. Two regions are strikingly anomalous to this pattern, the Eastern Highlands of Papua New Guinea and the Americas, both locations having 129V as the dominant allele (figure 2; Hardy *et al*. 2005). 127V has not fixed in any population studied. The E219K polymorphism (the only other change known to confer resistance to prion disease) is found at the highest frequency in Japan but also other populations in the Indian subcontinent and East Asia. In our study, one of these two established prion disease resistance polymorphisms was found in every population studied. Polymorphic sequence variants of *PRNP* are overwhelmingly non-synonymous: 1-octapeptide repeat deletion; M129V; G142S; N171S; E219K; M232R; and a further unpublished coding change, whereas only one synonymous change (A117A) achieves polymorphism status in these studies (non-synonymous : synonymous ratio 7 : 1). This ratio is in marked contrast to the ratio obtained by the comparison of ancestral human PrP with a range of primate PrPs (mean ratio non-synonymous : synonymous=0.39 : 1 from available great apes, old and new world monkeys (Schätzl *et al*. 1995)). The marked contrast in these ratios is statistically significant for all but the most closely related great apes with virtually no divergence from human PrP (chimpanzee and gorilla not significant, *n*=25 primate species (Schätzl *et al*. 1995)). These data suggest that the strong purifying selection, which has presumably resulted in the conservation of PrP coding sequence among primates, has relaxed or changed in recent human evolutionary history. Of particular note, an excess of human coding polymorphism is also consistent with balancing selection.

## 4. *PRNP* gene structure

The pattern of genetic diversity at a gene allows inference about its evolutionary history, in the context of the demography of the population analysed. Two papers concern *PRNP* gene genealogy. In 2003, we reported that the European *PRNP* gene genealogy is characterized by two highly divergent clades, representing 129V and 129M, a bimodal distribution of pairwise mutational differences and a skew in the allele frequency distribution to high-frequency polymorphism (Mead *et al*. 2003). However, a more recent report has challenged this finding, describing a star-shaped gene genealogy, a unimodal distribution of pairwise mutational differences and a skew in the allele frequency distribution favouring low-frequency polymorphism (Soldevila *et al*. 2005). Our analysis was interpreted as consistent with the episodes of balancing selection in recent human history (less than 500 000 years); although the interpretation of the more recent analysis was complex without clear signals, importantly, these authors rejected the balancing selection hypothesis, arguing that ascertainment bias had confounded the earlier results.

**Figure 3:**
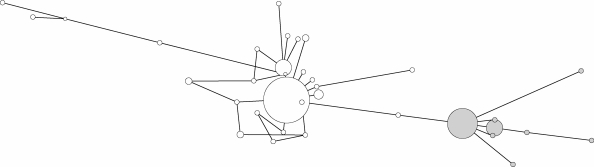
*PRNP* gene genealogy derived from the resequencing of 4.7 kb in 94 unrelated European individuals for part of the intron and exon 2, identifying 24 single nucleotide polymorphisms (with Network 4.5.0.0). Haplotypes were inferred from pedigree data. Discs are shown proportional to haplotype frequency (open, 129M; filled, 129V) and distances are proportional to the number of mutations separating haplotypes.

We resequenced the open reading frame and two regions of the adjacent intron of *PRNP* in 94 individuals from the Centre d'Etude Polymorphisme Humain (CEPH) family collection totalling 4.7 kb of sequence (figure 3). Our sequencing was conducted to fully ascertain rare variants in the sample. We found a nucleotide diversity of 0.0011, 24 segregating sites excluding octapeptide-repeat polymorphism and a Tajima's D of +0.80 (positive values in this statistic reflect an excess of high-frequency polymorphism). Although this finding was not significant in the context of the standard neutral model of evolution (which among other things assumes constant population size), when compared with empirical data of Stephens *et al*. (2001), the finding was significant at the 95 per cent level. The same summary statistic was also calculated for the available genotype data in a number of global populations. These cannot be compared meaningfully with theoretical population genetic models—unless ascertainment is modelled too—but a comparison of the different datasets with each other remains meaningful. This was reported in the paper and we found similar behaviour of the test statistic for the set of polymorphisms in different populations (these should not, however, have been mentioned in comparison to the resequencing data of Stephens *et al*. 2001). Our evidence indicates that the deepest genealogical split at *PRNP* is caused by the M129V polymorphism, and that ascertainment did not impact on our conclusions regarding gene genealogy, pairwise mutational differences and allele frequency skew. Soldevila and colleagues resequenced the entire *PRNP* exon 2 (2.4 kb) in 174 individuals from different world populations. Tajima's D in these populations ranged from −0.6 to −1.64 (not statistically significant), and nucleotide diversity was 0.00031 with 18 SNPs detected (Soldevila *et al*. 2005). We suspect that the different regions and length of resequencing (4.7 kb intron and open reading frame versus 2.4 kb exon 2) and the particular populations analysed (CEPH versus global diversity panel) contributed to the differences observed between analyses.

## 5. Conclusions

The use of genetic analysis to probe questions of human history and evolution is of considerable general interest. Others' and our own research in the Eastern Highlands province of Papua New Guinea have documented the powerful susceptibility and resistance effects of genotypes at codon 129 of *PRNP* in kuru. There is compelling evidence that the kuru epidemic imposed strong balancing selection on the Fore linguistic group and its neighbours exposed to kuru. These analyses afford a unique opportunity for individuals to be stratified by prion exposure and their resulting disease and incubation time, resulting in strong correlations with *PRNP* genotypes. In a global context, the EHP is extraordinary in its high frequency of 129V, bucking a global reducing cline towards the Far East. We have proposed that the remarkable number of coding polymorphisms in human populations and *PRNP* gene genealogy is consistent with the action of evolutionary processes in human history similar to those documented in the Fore.

## Figures and Tables

**Table 1:** *PRNP* codon 129 genotypes in susceptibility-stratified groups from EHP. (The *p* values are given for 3×2 (*Χ*^2^-tests, 2 d.f.) cross-tabulations. ‘n.s.’ denotes not significant.)

group	susceptibility	*n*	MM	MV	VV	%MM	%MV	%VV	*p* versus young modern Fore
kuru children	high	48	22	12	14	0.46	0.25	0.29	7.96×10^−5^
kuru adult	uncertain	94	12	69	13	0.13	0.73	0.14	7.66×10^−5^
kuru long incubation time	low	10	1	8	1	0.10	0.80	0.10	n.s.
elderly women (born before 1950)	low	125	16	86	23	0.13	0.69	0.18	0.001
elderly men (born before 1960)	moderately low	205	34	111	60	0.17	0.54	0.29	n.s.
young modern Fore	neutral	282	52	136	94	0.18	0.48	0.33	–
young from outside kuru region	neutral	631	145	311	175	0.23	0.49	0.28	n.s.
